# ACTG A5409 (RAD-TB): Study Protocol for a Phase 2 Randomized, Adaptive, Dose-Ranging, Open-Label Trial of Novel Regimens for the Treatment of Pulmonary Tuberculosis

**DOI:** 10.21203/rs.3.rs-5931694/v1

**Published:** 2025-03-26

**Authors:** Linda Harrison, Gustavo E Velasquez, Russell R Kempker, Marjorie Z Imperial, Eric Nuermberger, Susan E Dorman, Elisa Ignatius, Janeway Granche, Patrick PJ Phillips, Jennifer Furin, Eunsol Yang, Colleen Foley, Shawn Chiambah, Rochelle Rogers, Austin Van Grack, Jhoanna Roa, Justin Shenje, Sandy Nerette, Cecilia Kanyama, Rachel Bakyayita Kyeyune, Alberto Mendoza-Ticona, William Murtaugh, Salah Foraida, Melanie Goth, Andrew Vernon, Kelly E Dooley, Radojka M Savic

**Affiliations:** Harvard University T H Chan School of Public Health; UCSF: University of California San Francisco; Emory University; UCSF: University of California San Francisco; Johns Hopkins University; Medical University of South Carolina; Johns Hopkins University; Harvard University T H Chan School of Public Health; UCSF: University of California San Francisco; Case Western Reserve University; UCSF: University of California San Francisco; Frontier Science Foundation; National Institute of Allergy and Infectious Diseases; National Institute of Allergy and Infectious Diseases; Social & Scientific Systems Inc; Social & Scientific Systems Inc; University of Cape Town; GHESKIO; UNC Project-Malawi; Seattle Children’s Research Institute; SES: Socios En Salud Sucursal Peru; UCLA: University of California Los Angeles; Global Alliance for TB Drug Development: TB Alliance; National Institute of Allergy and Infectious Diseases; National Institute of Allergy and Infectious Diseases; Vanderbilt University Medical Center; UCSF: University of California San Francisco

**Keywords:** tuberculosis, drug-susceptible, platform trial, bedaquiline, pretomanid, linezolid, TBI-223, sutezolid, time to positivity, early efficacy, randomized controlled trial

## Abstract

**Background:**

The standard of care (SOC) treatment for drug-susceptible pulmonary tuberculosis (DS-TB) consists of isoniazid, rifampicin, pyrazinamide, and ethambutol (HRZE). New treatment regimen options for DS-TB are needed as HRZE is long in duration (6 months), associated with frequent adverse events, unforgiving of adherence lapses, and complicated by rifamycin-based drug-drug interactions. The recent resurgence of TB drug development, particularly in the context of drug-resistant TB, offers promise for additional regimens for persons with DS-TB, provided they are sufficiently effective and well-tolerated. We spotlight wave 1 of the RAD-TB platform trial (ACTG A5409, NCT06192160) that will investigate new chemical entities for the treatment of DS-TB.

**Methods:**

In wave 1 of the RAD-TB platform, adult participants initiating treatment for DS-TB will be randomized to SOC (HRZE, Arm 1) or one of five experimental arms for the 8-week intensive phase. The experimental treatment arms will consist of a bedaquiline and pretomanid backbone (BPa) in combination with one of three oxazolidinones. Arm 2 will study linezolid (BPaL) at a dose of 600 mg daily, Arms 3A and 3B will study TBI-223 at 1200 mg and 2400 mg daily, respectively, and Arms 4A and 4B will study sutezolid at 800 mg and 1600 mg daily, respectively. The primary efficacy objective is to compare sputum culture time to positivity (TTP) slope over the first 6 weeks of treatment for each experimental treatment arm to SOC. The primary safety objective is to compare new Grade 3 or higher adverse events over the first 8 weeks of treatment for each experimental treatment arm to SOC. After the intensive phase, all participants will receive the standard isoniazid and rifampicin (HR) continuation phase for 18 weeks and will be followed for 52 weeks after TB treatment initiation to assess long-term outcomes.

**Discussion:**

Wave 1 of the RAD-TB platform aims to identify the optimal oxazolidinone(s), with regard to both efficacy and safety, to combine with the BPa backbone for the treatment of DS-TB. Subsequent waves of this platform trial may add a fourth drug to the regimen, study new diarylquinolines to substitute for bedaquiline, or study novel agents from other TB drug classes.

**Trials registration:**

ClinicalTrials.gov NCT06192160. Registered on January 5, 2024, https://clinicaltrials.gov/study/NCT06192160

## Administrative information

Note: The numbers in curly brackets in this protocol refer to SPIRIT checklist item numbers. The order of the items has been modified to group similar items (see http://www.equator-network.org/reporting-guidelines/spirit-2013-statement-defining-standard-protocol-items-for-clinical-trials/). SPIRIT checklist items {26a}, {26b}, {11d}, {16b}, {16c}, {17a}, {17b}, {18a}, {18b}, {19}, {27}, {33}, {31c}, {5d}, {21a}, {23}, {25}, {31a}, {29}, {24}, {32} and {28} are solely covered in the Supplemental Appendix.

**Table:** 

Title {1}	A Phase 2 Randomized, Adaptive, Dose-Ranging, Open-Label Trial of Novel Regimens for the Treatment of Pulmonary Tuberculosis (RAD-TB)
Trial registration {2a and 2b}	ClinicalTrials.gov registration number NCT06192160
Protocol version {3}	Version 2.0 as of March 21, 2024
Funding {4}	The trial is funded by the Division of AIDS (DAIDS), National Institute of Allergy and Infectious Diseases (NIAID), National Institutes of Health (NIH) under Award Numbers UM1 AI068634, UM1 AI068636, UM1 AI106701 and UM1 AI179699.
	Some RAD-TB wave 1 drugs will be donated or provided by the Global Alliance for Tuberculosis Drug Development ("TB Alliance").
Author Details {5a}	See title page
Name and contact information for the trial sponsor {5b}	Division of AIDS (DAIDS), National Institute of Allergy and Infectious Diseases (NIAID) Melanie Goth, Medical Officer at DAIDS, melanie.goth@nih.gov
Role of sponsor {5c}	DAIDS participated in the design of the RAD-TB trial and reviewed and approved the protocol prior to initiation. Additionally, TB Alliance reviewed the protocol prior to initiation. The content of publications of the RAD-TB trial are solely the responsibility of the authors and do not necessarily represent the official views of DAIDS, TB Alliance, or the institutions with which the authors are affiliated.

## Introduction

### Background and Rationale {6a}

New innovations in the treatment of drug-susceptible pulmonary tuberculosis (DS-TB) are urgently needed to shorten treatment duration, enhance outcomes, and provide options for people who cannot tolerate standard therapy. The current standard of care (SOC) treatment for DS-TB was developed over 40 years ago (1–3). It is six-months in length consisting of a two-month intensive phase of isoniazid (INH, H), rifampicin (RIF, R), pyrazinamide (Z), and ethambutol (E), followed by a four-month continuation phase of INH and RIF [HRZE] (4). The HRZE regimen is generally effective but often needs to be prolonged beyond six months in persons with cavitary lung disease (5), is associated with low completion rates in some groups (6), is unforgiving of modest adherence lapses (7), can cause gastrointestinal, liver, eye, skin, and hypersensitivity adverse events (8–10), and is complicated by rifamycin-based drug-drug interactions (11, 12). New drugs and regimens for DS-TB are needed to achieve a success rate of more than 90%, meet key priorities of the Global Plan to End TB (13), and provide better options for both TB providers and patients that more closely align with target regimen profiles set forth by the World Health Organization (WHO) (5).

In the context of drug-resistant TB, there have been significant advances in regimen development resulting in the registration of bedaquiline (B) and pretomanid (Pa), which when given in combination with linezolid (LZD, L) [BPaL], achieved a 90% treatment success rate in the Nix-TB study (NCT02333799) in six months (14, 15) but resulted in adverse events from LZD-related mitochondrial toxicity in a majority of participants, most commonly later in treatment (after the first eight weeks). A follow-on dose- and duration-ranging trial of the LZD component of BPaL, ZeNix (NCT03086486), showed that a lower starting dose of LZD at 600 mg daily resulted in a similar treatment success rate (91%), with fewer participants experiencing treatment-limiting anemia, thrombocytopenia, and neuropathy (16). Mouse models have shown that combinations like BPaL that include a diarylquinoline, nitroimidazole, and oxazolidinone, respectively, are highly efficacious with the oxazolidinone being a significant contributor to efficacy (17–20). BPaL has not been evaluated in clinical trials among persons with DS-TB, and descriptions of its use in persons with DS-TB are limited (21, 22). Studying BPaL in DS-TB is important scientifically since, beyond providing a new treatment option for those with rifamycin-intolerant DS-TB, differences in populations who acquire DS-TB versus DR-TB and in the biology of rifamycin-susceptible versus rifamycin-resistant *Mtb* strains may lead to varying treatment responses (23–27). Knowledge of the efficacy of BPaL in DS-TB will additionally help to define a pan-TB regimen in the future.

While microbiologic and clinical outcomes with BPaL are excellent, mitochondrial toxicities associated with LZD, such as optic and peripheral neuropathy and myelosuppression, are a concern. These toxicities are related to cumulative exposure to the drug and limit our ability to use LZD safely beyond the first 8 weeks of treatment. Other oxazolidinones are in development that are likely to have a lower risk of side effects, as some have inhibitory concentrations against mitochondrial protein synthesis that are significantly higher than LZD. One example, TBI-223, recently completed phase 1 testing (28), had a superior toxicity profile compared to LZD in toxicology studies, and had comparable efficacy when replacing LZD in combination with a diarylquinoline and Pa in mouse models (29, 30). Secondly, sutezolid (SZD, S) has superior potency compared to LZD *in vitro* (31) and has demonstrated greater efficacy when administered alone and in combination with BPa in BALB/c mice (17–20). BALB/c mouse model data indicate that SZD together with BPa outperforms HRZE in bactericidal activity and probability of relapse at a dose of 50 mg/kg daily, which is equivalent to 600–800 mg daily in humans assuming comparable protein binding. SZD completed phase 2A early bactericidal activity (EBA) testing (32, 33), and in the SUDOCU phase 2B trial SZD doses of 600 mg daily, 1200 mg daily, 600 mg twice daily and 800 mg twice daily in combination with bedaquiline, delamanid, and moxifloxacin were investigated. Preliminary safety data suggest there were no dose-limiting safety issues, and pharmacokinetic-pharmacodynamic (PK-PD) analyses suggested there was an exposure-response relationship (34).

With multiple new chemical entities in the TB drug pipeline (35, 36), several trials networks and consortia are planning studies of new multidrug regimens for participants with DS-TB [CRUSH-TB (37), PAN-TB (38), PanACEA (39), UNITE4TB (40)], including our own network Advancing Clinical Therapeutics Globally for HIV/AIDS and Other Infections [ACTG] (41). The ACTG will conduct the novel ‘Randomized, Adaptive, Dose-Ranging, Open-Label Trial of Novel Regimens for the Treatment of Pulmonary TB’ (RAD-TB) (ACTG A5409, NCT06192160) platform trial (42). This paper spotlights wave 1 of the RAD-TB platform, where next-generation oxazolidinones in combination with BPa for the treatment of DS-TB are the primary focus.

### Trial Design {8}

RAD-TB is a phase 2, open-label, randomized controlled trial with an adaptive design, evaluating new regimens for the treatment of DS-TB. The trial utilizes a platform protocol that allows for future concurrently randomized treatment regimens to be added in subsequent waves after participants have completed, and outcomes have been evaluated for, the current wave.

#### Wave 1

**Figure 1:**
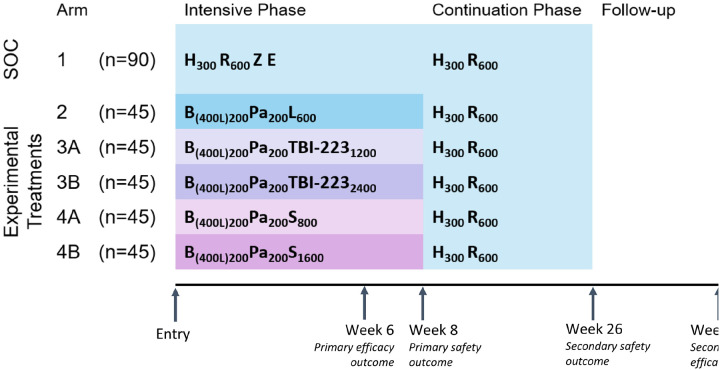
RAD-TB (ACTG A5409) Wave 1 Platform Trial Design Schematic

In wave 1 of the RAD-TB platform trial, participants with DS-TB will be randomized to one of six arms (Fig. 1). The first two arms of wave 1 will consist of an 8-week SOC HRZE intensive phase (Arm 1) and an 8-week BPaL intensive phase (Arm 2). In Arms 3A and 3B, LZD will be replaced in the BPa combination with one of two different doses of TBI-223 (1200 mg and 2400 mg daily, respectively). Our current mechanistic PK-PD dose-response model suggests that a TBI-223 daily dose of 1200 mg or 2400 mg will provide responses in combination with BPa that are similar to LZD (29). In Arms 4A and 4B, LZD will be replaced in the BPa combination by one of two different doses of SZD (800 mg and 1600 mg daily, respectively). Our translational model indicates that human SZD doses of at least 800 mg daily will be similar to or better than LZD in combination with BPa.

Wave 1 will enroll a planned 315 participants concurrently randomized to one of the six arms. Twice as many participants will be randomly allocated to the SOC HRZE arm (Arm 1, n = 90) compared to the five experimental treatment arms (Arms 2, 3A-B and 4A-B, n = 45 each, or 225 in total). This will ensure a stable within-trial SOC comparison arm. Participants will be treated for a total of 26 weeks; consisting of the 8-week intensive experimental phase followed by an 18-week SOC HR continuation phase. Primary efficacy and safety outcomes will be measured at 6 and 8 weeks after treatment initiation, respectively, and all participants will be followed for 52 weeks post-randomization to assess long-term outcomes.The first 20 participants randomized to each of the experimental treatment arms (Arms 2, 3A-B, 4A-B) who consent will undergo intensive sampling for PK analysis, and all experimental arm participants will undergo sparse PK sampling.

### Objectives {7}

#### Co-Primary Objectives

1. To compare mycobacteria growth indicator tube (MGIT) liquid culture time to positivity (TTP) slope over the first 6 weeks of treatment for each experimental treatment arm to the SOC arm.
2. To compare new Grade 3 or higher adverse events (AEs) over the first 8 weeks of treatment for each experimental treatment arm to the SOC arm.

### Secondary Objectives

- To compare time to stable culture conversion by MGIT liquid culture by week 8 for each experimental treatment arm to the SOC arm.
- To compare MGIT liquid culture TTP slope over the first 8 weeks of treatment for each experimental treatment arm to the SOC arm.
- To compare new Grade 3 or higher AEs over 26 weeks of treatment for each experimental treatment arm to the SOC arm.
- To compare discontinuations of anti-TB drugs for any reason prior to 8 and 26 weeks of treatment for each experimental treatment arm to the SOC arm.
- To determine the dose- and exposure-response relationships between experimental drug estimated PK parameters with safety and efficacy.
- To compare a composite of efficacy and safety outcomes using a risk-benefit approach for each experimental treatment arm to the SOC arm.
- To compare MGIT liquid culture TTP slope over the first 6 weeks of treatment for Arms 3A-3B and Arms 4A-4B compared to Arm 2 (BPaL).
- To compare durable cure defined by 52 weeks after treatment initiation in each experimental treatment arm to the SOC arm.

## Methods: Participants, interventions, and outcomes

### Study Setting {9}

The RAD-TB platform trial will be conducted at international sites of the ACTG trials network located in 13 countries in Africa, Asia, and South America.

### Eligibility Criteria {10}

#### Inclusion Criteria

Overall, the RAD-TB platform trial will recruit adult participants (≥18 years) who have active pulmonary DS-TB and are initiating a course of therapy. Key inclusion criteria are

- Pulmonary TB identified by a sputum specimen within 7 days of entry that is positive for *Mycobacterium tuberculosis* (*Mtb*) and has a semiquantitative result of medium or high by Xpert
- No prior history of TB treatment within the last five years
- Documentation of susceptibility to INH and RIF
- For individuals with HIV, a CD4 count ≥ 100cells/mm^3^ and currently or planned to be treated with dolutegravir-based antiretroviral therapy
- Normal laboratory values, a Karnofsky score ≥ 60, intention to follow contraception requirements, and ability and willingness to provide informed consent

#### Exclusion Criteria

Participants with more than 7 days of treatment for the current episode of active TB, extrapulmonary TB, Grade 2 or higher peripheral neuropathy, a QTcF interval > 450 ms, a weight < 35 kg, or a history of congenital QT prolongation, heart failure, hypothyroidism, bradyarrhythmia, or torsades de pointes will be excluded. Because the safety and efficacy of experimental compounds in this early phase trial have not yet been sufficiently established, individuals who are currently pregnant or breastfeeding will be excluded. See the Supplemental Appendix for a full list of the inclusion and exclusion criteria.

### Interventions

#### Explanation for choice of comparator {6b}

The study interventions are given during the first 8 weeks of TB treatment and the primary comparator regimen is HRZE SOC (Arm 1). HRZE was chosen as the primary comparator since it is the WHO-recommended regimen for pulmonary DS-TB, thus allowing for a direct comparison of the experimental treatment regimens with the current SOC. This trial also utilizes a second comparator regimen in BPaL (Arm 2). While the primary analysis will compare experimental treatment regimens to HRZE, BPaL will secondarily be compared to the other experimental treatment arms and was chosen since it is a WHO-recommended regimen for DR-TB and its use as an additional internal comparator will enable more informative ranking and prioritization of regimens (See the section on ‘Methods for additional analyses’ {20b} below for more details). Specifically, comparison of the novel BPa-containing regimens with BPaL will allow for direct comparison of the safety and microbiologic activity of different oxazolidinones.

#### Intervention description {11a}

Control and experimental treatment regimens (weeks 1–8) in wave 1 of the RAD-TB platform are displayed in Fig. 1 and outlined below:

Arm 1: Isoniazid, Rifampicin, Pyrazinamide, Ethambutol [HRZE]
Arm 2: Bedaquiline, Pretomanid, Linezolid [BPaL]
Arm 3A: Bedaquiline, Pretomanid, TBI-223 (1200mg)
Arm 3B: Bedaquiline, Pretomanid, TBI-223 (2400mg)
Arm 4A: Bedaquiline, Pretomanid, Sutezolid (800mg)
Arm 4B: Bedaquiline, Pretomanid, Sutezolid (1600mg)

All drugs will be given once daily with the doses specified in Fig. 1 and will be provided by the study through week 8. Bedaquiline will be given with a loading dose of 400 mg daily for the first two weeks followed by 200 mg daily for six weeks. After week 8, through week 26, all participants will receive the HR continuation phase through their in-country national TB program (NTP) at doses shown in Fig. 1. Pyridoxine (vitamin B6) will be given with INH based on current local dosing guidelines.

### Criteria for discontinuing or modifying allocated interventions {11b}

#### Treatment Interruptions

Study participants will have up to 70 days (10 weeks) from entry to complete 56 doses (8 weeks) of experimental treatment. Any missed doses should be made up with the same combination of drugs that were missed. For all arms during the first 8 weeks, a partial missed dose, where some but not all study drugs in the assigned regimen were taken, will be considered a full missed dose and will need to be made up at the end of the 8-week experimental treatment period.

#### Treatment Discontinuation

Participants who develop a Grade ≥ 3 AE or toxicity thought to be secondary to study drugs or of unknown etiology must be discussed by the site investigator with the trial clinical management committee (CMC), will have all study-provided drugs permanently discontinued, and will be referred to the NTP for completion of their TB treatment according to local SOC. If study-provided drug is permanently discontinued, participants will still be followed through the 52-week visit. If a participant develops visual changes which are considered likely due to the oxazolidinone, then the oxazolidinone (and other study-provided drugs) will be permanently discontinued at any grade of presumed optic neuritis. Participants who become pregnant or begin breastfeeding during the study will be discontinued from study-provided drugs and referred to the NTP for the treatment of their TB according to local SOC, and to a prenatal or postnatal care program for management of their pregnancy or breastfeeding, respectively, according to local SOC. Full criteria for permanent and premature study treatment discontinuation are provided in the Supplemental Appendix.

#### Strategies to improve adherence to interventions {11c}

Directly observed therapy (DOT) will be performed throughout TB treatment. Each site must follow local TB guidelines about DOT. All drugs must be taken orally, 7 days per week. At least five doses per week must be administered as DOT. Video DOT, use of community health workers, or other strategies used locally for delivering observed therapy are acceptable. Doses taken on weekends and on holidays may be under DOT or self-administered, as permitted by local TB guidelines. Data on adherence including pill intake will be recorded on standardized electronic case report forms (eCRFs). Participants with lower adherence (< 95%) will be provided counseling by the site. Additionally, all study participants will have adverse event counseling performed by study staff at entry, and at study weeks 1, 2, 3, 4, 6 and 8.

#### Provisions for post-trial care {30}

Participants who prematurely discontinue study treatment will be referred to their NTP or local clinic for treatment of their TB according to local SOC, but will be encouraged to continue on study, off study treatment, and receive all evaluations per the schedule of evaluations (SOE) through week 52 (Table 1). The composition of the treatment regimen once a participant is discontinued from the study will be at the discretion of the local clinician, with the trial CMC available as needed to advise.

### Outcomes {12}

#### Primary Efficacy Outcome Measure and Estimand

The primary efficacy outcome is measured by TTP from longitudinal MGIT liquid culture measurements at weeks 0, 1, 2, 3, 4 and 6 of treatment. The primary efficacy estimand is the difference in mean (experimental arm versus SOC) log_10_ TTP slope from longitudinal MGIT liquid culture measurements over the first 6 weeks of treatment.

#### Primary Safety Outcome Measure and Estimand

The primary safety outcome measure is a new Grade 3 or higher AE through week 8 of treatment. The primary safety estimand is the difference in cumulative proportion (experimental arm versus SOC) of individuals having at least one new Grade 3 or higher AE by week 8 of treatment.

#### Secondary Outcome Measures

The secondary outcome measures are, as follows:

- Stable sputum culture conversion by week 8 as measured by culture negative status via MGIT liquid culture at two consecutive measurements.
- TTP slope from longitudinal MGIT liquid culture measurements over the first 8 weeks of treatment.
- New Grade 3 or higher AE through week 26 of treatment.
- Permanent discontinuation of study-provided anti-TB drugs due to any reason prior to week 8 of treatment.
- Permanent discontinuation or temporary discontinuation for ≥ 3 days of at least one anti-TB drug due to any reason prior to week 8 of treatment.
- Permanent discontinuation of at least one anti-TB drug due to any reason prior to week 26 of treatment.
- A composite of stable culture conversion at week 6 of treatment and no new Grade 3 or higher AE through week 8.
- Durable cure by 52 weeks after treatment initiation.

#### Other Outcome Measures

Other outcome measures are, as follows:

- Projected hazard ratio comparing time to stable culture conversion for each experimental treatment arm to SOC.
- Sputum ribosomal RNA synthesis (RS) ratio over the first 8 weeks of treatment and at 26 weeks of treatment (43).
- MGIT liquid culture results at weeks 1, 2, 3, 4, 6, and 8 of treatment.

#### Participant timeline {13}

Following informed consent, individuals will be screened for the trial to determine if inclusion and exclusion criteria are met. Eligible participants will be randomized to a treatment assignment at entry. Post-entry, scheduled evaluations will take place weekly until week 4, every two weeks until week 12, then at weeks 16, 20, 26 and 52. Additionally, unscheduled visits will occur at premature treatment or study discontinuation, or when a possible poor treatment response is suspected at or after week 16. Table 1 displays the planned evaluations at each visit [See Supplemental Fig. 1 for the SPIRIT figure]. Sputum for mycobacterial culture in liquid media will be collected at each visit with two sputum samples collected at entry and weeks 4, 6 and 8. At participating sites, open-ended qualitative interviews will be conducted in consenting participants to explore patient preferences for treatment regimens using systematic qualitative methods (44).

#### Sample Size {14}

The sample size in wave 1 will be 45 participants in each experimental treatment arm and 90 participants in the SOC arm (Arm 1). Assuming that 10–12% of participants will undergo late exclusion, withdraw from the study, or have several missing TTP measurements, we based the power simulation on 80 evaluable participants in the efficacy set in the SOC arm, and 40 in each experimental treatment arm. Using longitudinal liquid culture data from the HRZE arm of a recent large, international Phase 3 trial (TBTC Study 31/ACTG A5349, NCT02410772) (45), we estimated a baseline (intercept) TTP of 0.91 log_10_ days and a TTP slope of 0.13 log_10_ days per week via a linear mixed-effects model with an additive random intercept (SD = 0.101) and slope (SD = 0.353) plus a multiplicative random error (SD = 0.161). Using these estimates, TTP outcomes over 6 weeks were simulated (1,000 replicates) for a SOC and experimental treatment arm with right-censoring of TTP greater than 42 days. For each simulated dataset, a linear mixed-effects model on the log_10_ TTP scale accounting for TTP censoring was fit.

**Figure 2:**
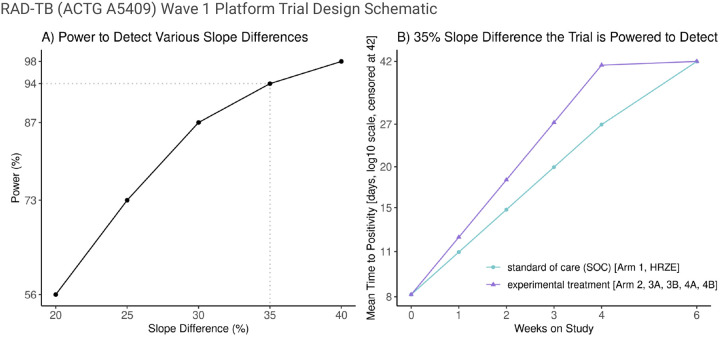
Power for the Primary Efficacy Analysis

Based on this simulation, the trial will provide over 90% power to detect a difference in log_10_ TTP slopes of at least 35% with a two-sided 5% significance level for each experimental treatment arm compared to SOC (Fig. 2A). As an example, if the average baseline TTP is 0.91 log_10_ days = 8.1 days and the TTP increase is 0.13 log_10_ days per week in the SOC arm, then by week 4 the TTP will be an average of 27 days for the SOC arm. The trial will have over 90% power to detect a 35% increase in slope on the log_10_ TTP scale (Fig. 2B). This translates to being powered to detect an average TTP of 41 days by week 4 for an experimental arm. No adjustment for multiple testing is planned.

#### Recruitment {15}

Persons presenting to an international ACTG study site with at least one sputum specimen positive for *Mtb* by Xpert MTB/RIF Ultra at a medium or high semiquantitative level will be invited to screen for the study. The details of the study will be carefully discussed, and the candidate will be asked to read and sign the informed consent form (ICF). Those who agree will enter screening and will be assessed for eligibility by a local study investigator. If they meet all inclusion and none of the exclusion criteria they will be enrolled. Recruitment to wave 1 is anticipated to take approximately 12 months.

### Assignment of interventions: allocation

#### Sequence generation {16a}

In wave 1, participants will be randomly assigned to Arm 1 (SOC) or to one of the five concurrently enrolling experimental treatment arms (Arms 2, 3A-B or 4A-B) in a 2:1:1:1:1:1 ratio. Randomization will be conducted using permuted blocks within each of two strata defined by a medium versus high semiquantitative *Mtb* result by Xpert MTB/RIF Ultra. Additionally, randomization will be dynamically balanced by site.

### Statistical Methods

#### Statistical methods for primary and secondary outcomes {20a}

In wave 1, all comparisons will primarily be made between concurrently randomized experimental treatment and the SOC arm. Using TTP measurement from longitudinal MGIT liquid culture measurements at weeks 0, 1, 2, 3, 4 and 6 of treatment, we will estimate the difference in the mean (experimental arm versus SOC) log_10_ TTP slope over the first 6 weeks of treatment. This primary efficacy outcome will be analyzed in the efficacy set of all randomized participants who take at least one dose of study treatment who do not undergo late exclusion due to either a negative baseline culture or drug resistance by baseline phenotypic testing. Table 2 provides the full estimand and analysis details [per the ICH addendum (46, 47)].

For the primary safety outcome measure, the difference in the cumulative proportion (experimental arm versus SOC) of an individual having at least one new grade 3 or higher AE by week 8 of treatment will be estimated. This outcome will be analyzed in the safety set of all randomized participants who take at least one dose of study treatment (See Table 2 for a full specification of the planned analysis). Details of the planned analysis for the secondary outcome measures are in the Supplemental Appendix.

#### Interim Analyses {21b}

We devised an interim safety stopping guideline to allow early discontinuation of experimental treatment arms if they are unlikely to meet a safety guideline at the end of wave 1 (specified in the next section). The interim safety stopping guideline will be assessed when approximately half the participants in the wave have been randomized and reached 8 weeks of follow-up. At this time the observed difference in the cumulative proportion of new Grade 3 or higher AEs by week 8 for each experimental treatment arm compared to the SOC arm will be estimated. If there is a > 30% higher observed probability of a new Grade 3 or higher AE through week 8 compared to SOC, the independent interim review committee will consider stopping that experimental treatment arm. For example, if 9 participants out of 22 (41%) experience a new grade 3 or higher AE on an experimental treatment arm compared to 4 out of a 45 (9%) on the SOC arm, the interim safety stopping guideline will be met. This safety stopping guideline is informed by assuming that approximately 10% of participants in the SOC arm will experience a new Grade 3 or higher AE by week 8 (based on data from S31/A5349 (45)). Given this, when the true difference between an experimental treatment arm and the SOC arm is 40%, there is a high chance (~ 81%) of meeting the stopping guideline. Whereas, when there is truly no difference between an experimental treatment arm and the SOC arm there is a very low chance (< 1%) of meeting the stopping guideline (See Table 3). Additionally, if the interim safety stopping guideline is met there is a high chance the regimen will be unlikely to be considered at the end of wave 1 for further development based on the safety guideline for ranking regimens provided in the next section. Wave 1 will also undergo additional regular safety reviews (see details in the Supplemental Appendix).

### Methods for additional analyses {20b}

When ranking the TB regimens in wave 1 and deciding which regimens to move forward into subsequent waves of the RAD-TB platform (with four-drug regimens, for example), the totality of internal trial evidence, as well as evidence external to the trial, will be considered. A regimen that has a > 15% higher observed probability of a new grade 3 or higher AE through week 8 compared to SOC will be unlikely to be considered. Once a regimen passes this safety guideline, the primary efficacy analysis (TTP slope over the first 6 weeks of TB treatment) will be considered to rank regimens, along with the regimen’s risk-benefit profile, the regimen’s overall safety and tolerability profile, and PK parameters. Additionally, the mean difference in TTP slopes and associated 95% CIs over the first 6 weeks of treatment for Arms 3A-3B and Arms 4A-4B will be compared to Arm 2.

#### Methods in analysis to handle protocol nonadherence and any statistical methods to handle missing data

The analysis for the primary efficacy and safety outcomes will primarily use a treatment policy approach (46) for intercurrent events via an intention-to-treat analysis strategy (See Table 2). Supplemental analyses will consider a while-on-treatment approach for non-minor treatment changes. Participants with a missing TTP will be considered missing at random given observed TTP measurements and the randomized arm. Participants with an inability to produce sputum with or without induction will be assumed to have a TTP > 42 days and to be culture negative.

### Oversight and Monitoring

#### Adverse event reporting and harms {22}

Severity of AEs will be graded according to the DAIDS Table (48), except for creatinine, peripheral neuropathy, QT, visual acuity, and color vision, which will use trial-specific severity grading scales. Throughout trial follow-up, all Grade ≥ 1 non-laboratory AEs, Grade ≥ 2 laboratory AEs, all AEs of special interest, all AEs that lead to a change in study treatment, and any AEs meeting the serious AE (SAE) definition or expedited AE (EAE) reporting requirement (49) will be recorded on the eCRFs within 3 days. AEs of special interest include those related to oxazolidinone use including peripheral neuropathy, optic neuritis, and cytopenias as well as QT prolongation and increased transaminases.

## Discussion

The ultimate aim of the RAD-TB platform trial is to build an optimized four-drug regimen for the treatment of DS-TB that will have superior efficacy compared to HRZE, the potential to shorten treatment duration, and safety and tolerability that is at least as good as HRZE. Currently, clinicians caring for patients with DS-TB do not have many trial-proven alternatives. Here, we have described the study protocol for wave 1 of the RAD-TB platform trial. Wave 1 will consist of six randomized treatment arms to identify the best oxazolidinone(s) to combine with the BPa backbone. Subsequent waves of the RAD-TB platform trial may include further refinement of successful regimen(s) identified in wave 1 to construct a four-drug regimen for DS-TB; this may include addition of a fourth drug or dose-ranging evaluations of novel agents from different drug classes.

The study design of the RAD-TB platform trial is innovative in at least four ways. First, RAD-TB includes a strategy to rank the regimens within a wave before advancing regimens to the next wave of the platform. At the end of a wave, a safety guideline will be applied before assessment of efficacy, followed by consideration of other factors such as the regimen’s risk-benefit profile and PK parameters. The wave has been designed to include an interim safety review at 50% information so that regimens that are unlikely to meet the end-of-wave safety guideline are stopped early. Second, RAD-TB includes an SOC comparator arm composed of the WHO-recommended treatment for DS-TB, HRZE (Arm 1) (4), as well as a second comparator of BPaL (Arm 2); a current WHO-recommended treatment for DR-TB (50). This allows for a within-trial comparison to a second comparator where only one component of the regimen will be modified relative to the experimental arms. For example, a within-trial comparison of TTP slope will be made between BPaL (Arm 2) and BPa with TBI-223 at 1200 mg daily (Arm 3A). The inclusion of BPaL will also provide a first-ever benchmark of BPaL head-to-head against HRZE in DS-TB. Third, the primary efficacy readout will use longitudinal liquid culture TTP measurements over the first 6 weeks of treatment. The TTP slope will be modeled using analysis techniques appropriate for right-censoring as well as for repeated measures within participants. This allows for an efficient assessment of efficacy in an early phase trial of new TB treatment combinations (51). Fourth, RAD-TB will conduct dose-finding within the same trial infrastructure with two arms for both TBI-223 (Arms 3A and 3B) and SZD (Arms 4A and 4B). This enables efficient determination of the optimal dose of these two oxazolidinones and assessment of dose-response.

RAD-TB puts forward a paradigm-changing advancement in TB drug development. Internally, we refer to RAD-TB as a ‘Phase 2A+’ design. Having completed Phase 1 testing, TBI-223 will bypass dedicated EBA monotherapy trials (‘Phase 2A’) in its FDA registration pathway. This has opened a new accelerated drug development pathway, which TBAJ-876 (52) has also followed. The RAD-TB design permits this through its rich sputum sampling in the intensive phase which, coupled with translational modeling predictions (53), will provide efficacy and safety data for TBI-223 that will stand in for the data generated in Phase 2A. And, as a dose-ranging study of combination therapy with follow-up through 52 weeks post-randomization, RAD-TB will serve the role of what is traditionally considered a Phase 2B trial for both TBI-223 and SZD. This is a welcome advance for TB drug development, where Phase 2A studies have been instrumental to the evaluation of the efficacy of single drugs — but are susceptible to false negatives where lack of robust EBA may be misconstrued as lack of contribution to multidrug therapy. A salient example is pyrazinamide, which has modest EBA but high sterilizing activity (54). Pyrazinamide was critical in shortening standard therapy for DS-TB from 9 to 6 months (1).

Wave 1 of the RAD-TB trial will generate critical performance and safety data on two novel, promising oxazolidinones, SZD and TBI-223, among persons with DS-TB. While LZD has become a cornerstone drug for the treatment of multidrug- and extensively drug-resistant TB (50) based on efficacy demonstrated in the Nix-TB, ZeNix, and TB-PRACTECAL trials (15, 16, 55), concerns remain regarding its high rate of AEs, especially when given for more than two months. LZD is associated with myelosuppression and peripheral neuropathy resulting in dose reduction or treatment interruption in many patients. AEs are related to the off-target binding of LZD to mammalian mitochondrial ribosome leading to the inhibition of mitochondrial protein synthesis. The narrow therapeutic window of LZD along with a scarcity of data among persons with DS-TB necessitates further study into alternative oxazolidinones. SZD is a thiomorpholine structural analogue of LZD that was developed alongside LZD but has taken a protracted path to being rigorously tested for TB treatment (56). A promising characteristic of SZD is its enhanced potency against *Mtb* including being more active in caseum (57). The minimum inhibitory concentrations of SZD against *Mtb* clinical isolates have been found to be three times lower than LZD and murine studies have found greater efficacy of BPa and SZD regimens (58) versus first-line drug-susceptible therapy and also versus BPa and LZD regimens (17). Additionally, the main metabolite of SZD, U-101603, which is more abundant than the parent compound, has activity against *Mtb* and appears more active against non-replicating bacteria. Early clinical studies have found SZD to be safe and have bactericidal activity when given for 14 days (31). TBI-223 is a newly developed oral oxazolidinone with high bioavailability and substantially reduced inhibition of mammalian mitochondrial protein synthesis. It was developed to optimize the efficacy and safety of oxazolidinone therapy. TBI-223 has activity against drug-susceptible and resistant *Mtb* isolates from all global lineages and against replicating and non-replicating *Mtb*. In a murine model, TBI-223 has demonstrated similar bactericidal and sterilizing ability to LZD (30). The main advantage of TBI-223 over LZD is its reduced potential for AEs given its low rate of mitochondrial protein synthesis inhibition. Utilizing available pre-clinical and clinical data for SZD as well as unpublished data for TBI-223, our translational pharmacology and modeling tools indicate that BPa with either SZD or TBI-223 will perform as well or better than LZD (29). Findings from RAD-TB will thus provide a critical direct comparison of the safety and efficacy of these two promising oxazolidinones, helping to define their role in treating TB.

In summary, RAD-TB is a platform trial studying early efficacy and safety of TB combination regimens within the ACTG trials network. Wave 1 of the RAD-TB platform will efficiently assess the best oxazolidinone(s) to use in combination with bedaquiline and pretomanid. Subsequent waves will build a safe and well-tolerated regimen that has the potential to be highly efficacious and reduce treatment length. The regimens efficiently identified by the RAD-TB platform will enable future studies of promising combinations that assess long-term outcomes in a large number of participants.

## Abbreviations

DS-TB: drug-susceptible tuberculosis
BPaL: bedaquiline, pretomanid, linezolid
HRZE: isoniazid, rifampicin, pyrazinamide, ethambutol
RAD-TB: Randomized, Adaptive, Dose-Ranging, Open-Label Trial of Novel regimens for the Treatment of Pulmonary Tuberculosis
